# Failure of daratumumab in the treatment of anti-synthetase syndrome: A case report

**DOI:** 10.1177/2050313X251336022

**Published:** 2025-04-29

**Authors:** Nam Nguyen, Valérie Leclair, Rayan Kaedbey, Marie Hudson

**Affiliations:** 1 Faculty of Medicine and Health Sciences, McGill University, Montreal, QC, Canada; 2 Division of Rheumatology, Lady Davis Institute for Medical Research, Jewish General Hospital, Montreal, QC, Canada; 3 Division of Hematology, Jewish General Hospital, Montreal, QC, Canada

**Keywords:** anti-synthetase syndrome, myositis, treatment, daratumumab

## Abstract

Anti-synthetase syndrome is a subset of idiopathic inflammatory myopathies, for which refractory disease can be difficult to treat. Daratumumab, an anti-CD38 monoclonal antibody primarily used in hematologic malignancies, has recently shown promise in very few case reports of refractory idiopathic inflammatory myopathies. We present a case of a 44-year-old man with anti-Jo1 anti-synthetase syndrome with concurrent anti-Ro52, in which myositis remained refractory to multiple immunosuppressants. Despite treatment with daratumumab, the patient did not respond and ultimately succumbed to infection. This case underscores the need for further research to better define the role of daratumumab in idiopathic inflammatory myopathies and identify predictors of treatment response.

## Introduction

Idiopathic inflammatory myopathies (IIM), including anti-synthetase syndrome (ASS), pose significant morbidity and mortality risks.^1^ While immunosuppression is the mainstay of treatment, optimal choices of agents for refractory cases remain uncertain.^1^ Daratumumab, primarily used in hematologic malignancies, has shown promise in the treatment of refractory IIM in case reports.^2–4^ In contrast, we report a case of an ASS unresponsive to daratumumab.

## Case presentation

**Figure 1.**
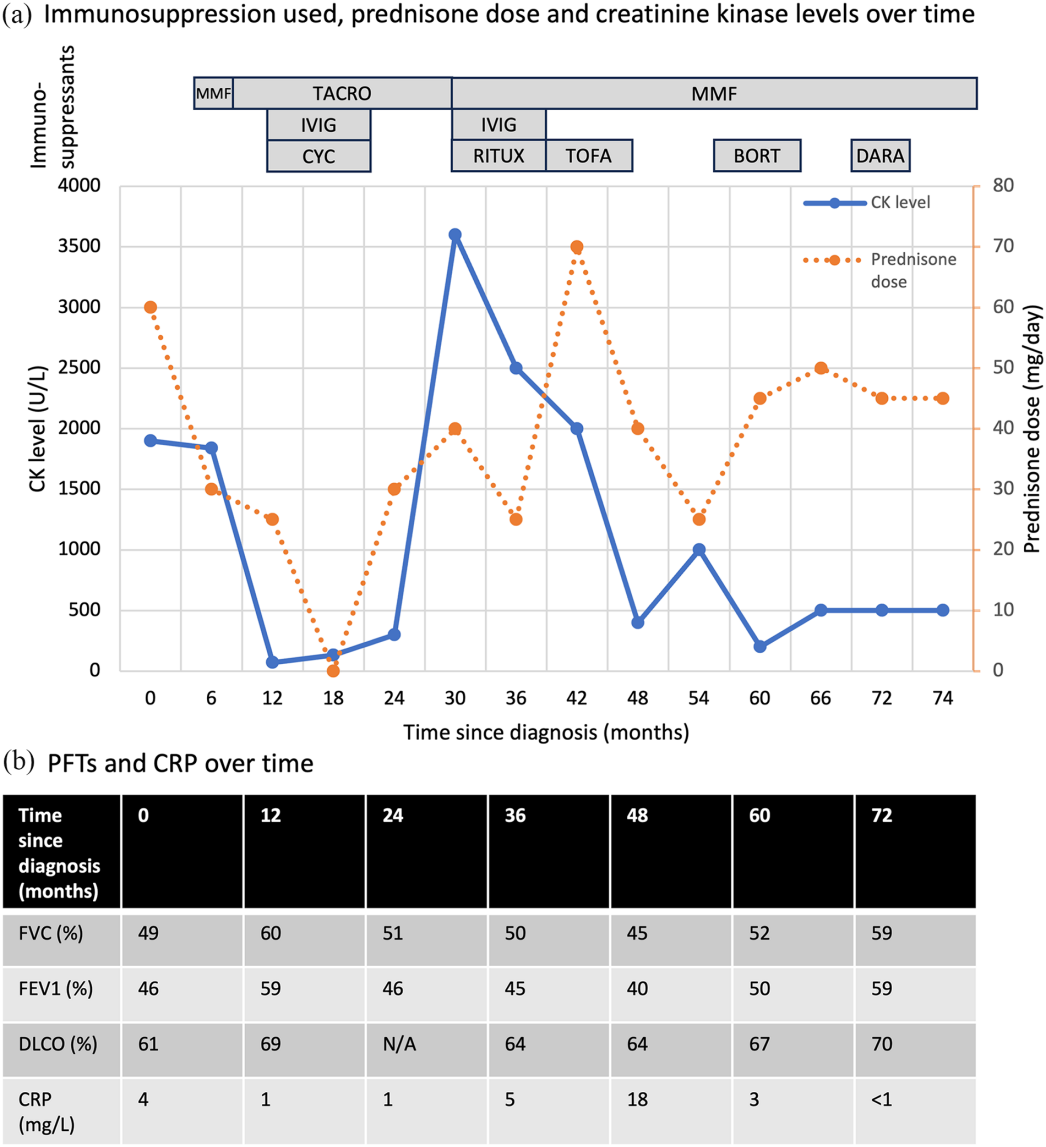
CK levels, prednisone dose, immunosuppressants used and pulmonary function over time. (a) The rectangles indicate the different immunosuppressants used over time. When prednisone was tapered, the myositis recurred with rising CK levels and prominent myalgias. (b) PFT values expressed in percentage predicted for age, sex, height and ethnicity. BORT: bortezomib; CK: creatine kinase; CYC: cyclophosphamide; DARA: daratumumab; IVIG: intravenous immunoglobulins; MMF: mycophenolate mofetil; RITUX: rituximab; TACRO: tacrolimus; TOFA: tofacitinib; CRP: C-reactive protein; PFT: pulmonary function test; FEV1: forced expiratory volume in 1 s; FVC: forced vital capacity; DLCO: diffusing capacity of the lung for carbon monoxide.

A 44-year-old Caucasian male known for factor V Leiden and metabolic syndrome presented with 3 months of dyspnea, cough, mechanic’s hands, myalgias and fevers. Initial investigations revealed an elevated creatine kinase (CK) level at 1900 U/L (normal range 42–396 U/L). Other investigations included C-reactive protein (CRP) 0.7 mg/L (normal range 0–10 mg/L), ferritin 153 µg/L (normal range 30–300 µg/L), lactate dehydrogenase 294 U/L (normal range 110–220 U/L), neutrophil:lymphocyte ratio 3.31 (normal range 0.78–3.53), myopathic changes on electromyography and organizing pneumonia on chest computed tomography. Pulmonary function tests (PFTs) revealed moderate restrictive disease (Figure 1(b)). Serologies were positive for anti-nuclear antibody (titer 1:160; pattern speckled and cytoplasmic), anti-Jo1 (high positive) and anti-Ro52 (medium positive) antibodies (Euroimmun, Lübeck, Germany). A cardiac magnetic resonance (CMR) scan was normal (troponin T 7 ng/L, normal range 0–15 ng/L). A muscle biopsy was consistent with an immune myopathy with perimysial pathology (IMPP).^5^

He was initially treated with prednisone 60 mg daily and mycophenolate mofetil (MMF) (Figure 1(a)). Given persistently elevated CK levels, he was then sequentially treated with tacrolimus, intravenous cyclophosphamide and intravenous immunoglobulins (IVIG), MMF, rituximab, tofacitinib and bortezomib. However, the myositis recurred each time steroids were tapered. A repeat CMR showed mild myocarditis (troponin I 23.1 ng/L, normal range 0–17.5 ng/L). A repeat muscle biopsy was again consistent with IMPP. Autologous hematopoietic stem cell transplant was considered, but he could not afford to relocate near a transplant center for peri-transplant care. Of note, fever was not a feature of his disease, CRP was generally normal and serial PFTs showed stable interstitial lung disease over time (Figure 1(b)). Plasma exchange was not used.

Given his persistently high steroid requirements (up to 0.5 mg/kg/day), daratumumab was initiated. Daratumumab was obtained by special permission from the hospital’s Pharmacy and Therapeutics Committee. He underwent 4-week cycles of weekly 1800 mg subcutaneous injections. The treatment course was complicated by bacterial pneumonia, but his pulmonary function remained stable. The treatment was stopped after five cycles due to lack of therapeutic effect, as the myositis recurred when the prednisone was decreased to 30 mg daily. He died a year later of SARS-CoV-1 pneumonia. A post-mortem review of the previous muscle biopsy specimens showed no cells positive for CD138, a marker for plasma cells.^6^ Written informed consent to report this case was obtained from the patient’s next of kin.

## Discussion

This case underlines the complexity of managing refractory IIMs and the limited understanding of their pathophysiology.^1^ Long-lived plasma cells, which are thought to be involved in the pathophysiology of refractory cases, are not targeted by conventional immunosuppressants such as rituximab, creating a pathway for disease resistance.^1,7^

Daratumumab is a monoclonal antibody against CD38, depleting several antibody-producing immune cells, including long-lived plasma cells.^7^ Daratumumab was shown in case reports to treat some autoimmune diseases, including three cases of refractory IIMs.^7^ Daratumumab induced remission in two cases of refractory MDA5-positive dermatomyositis.^2^ The first case contrasted with ours by the predominance of pulmonary over muscle involvement, the presence of CD38-positive plasma cells on flow cytometry (which we did not document), the shorter disease duration, the myositis response to other immunosuppressants and the recent use of rituximab.^2^ The second case also differed from ours by the pulmonary disease predominance and the shorter disease duration, as well as concomitant treatment with rituximab, tofacitinib and plasmapheresis.^4^ Daratumumab has also been reported to successfully treat a case of anti-signal recognition particle (SRP) IIM with severe muscle weakness.^3^ In that case, there was also recent administration of rituximab and concomitant use of methotrexate, tacrolimus and IVIG, making it more difficult to ascertain the response to daratumumab. The absence of plasma cells on the muscle biopsies in our case does not exclude the fact that these may have been present in his bone marrow. The main limitation in our case is the absence of a bone marrow biopsy to confirm the absence of long-lived plasma cells.

## Conclusion

Despite promising results, the failure of daratumumab in our case highlights the importance of further studies to confirm the efficacy of daratumumab in IIM, identify factors that could predict treatment response and study the effect of combination therapies.
